# Alpha-Lipoic Acid as a Means of Influence on Systemic Inflammation in Type 2 Diabetes Mellitus Patients with Prior Myocardial Infarction

**DOI:** 10.25122/jml-2020-0018

**Published:** 2020

**Authors:** Nataliia Valeriivna Altunina, Viktor Grigorovich Lizogub, Oleksandr Mykolayovych Bondarchuk

**Affiliations:** 1. Fourth Department of Internal Medicine, Bogomolets National Medical University, Kyiv, Ukraine

**Keywords:** coronary heart disease, non-Q-myocardial infarction, proinflammatory cytokines, anti-inflammatory effect, placebo-controlled studies

## Abstract

Patients with combined coronary heart disease and diabetes mellitus make up a growing segment of the population and require a comprehensive treatment approach. Patients with concurrent diabetes mellitus and coronary heart disease have a worse projection. Under these conditions, the incidence of recurrent myocardial infarction, early disability due to complications, and the risk of coronary death are increased. Therefore, the priority task is to find ways to optimize drug treatment of this category of patients, taking into account the impact of drugs on the pathogenetic links of coronary heart disease progression and the development of cardiovascular complications. One hundred twelve people were examined in the research. The patients had type 2 diabetes with a history of non-Q-myocardial infarction receiving oral antidiabetic therapy and basic therapy, including an ACE inhibitor, a β-blocker, a statin, and an antiplatelet agent. Analysis of the investigated parameters in the leading group after receiving alpha-lipoic acid for 4 months showed a significant decrease in the concentration of C-Reactive Protein, IL-6 and TNF-α. According to the results of our research, taking alpha-lipoic acid for 4 months in patients with type 2 diabetes who underwent non-Q-myocardial infarction reduced the activity of systemic inflammation and did not significantly affect the content of anti-inflammatory IL-10 in patients. In light of the above, it is of interest to administer alpha-lipoic acid to these patients, considering the positive effects of the agent such as antioxidant properties, vasorelaxation, positive metabolic profile, as well as an anti-inflammatory potential.

## Introduction

Among the causes of disability and mortality both in Ukraine and the world, coronary heart disease (CHD) occupies a leading position [1]. Among the many risk factors, diabetes mellitus (DM) is recognized as one of the most negative in terms of its impact on coronary heart diseases.

DM is associated with accelerated atherosclerosis progression [2, 3]; thus, atheromas in patients with diabetes contain more lipids, are more inflammatory and are characterized by a higher risk of thrombus formation than individuals without diabetes [4]. In this regard, vascular endothelial dysfunction, the peroxidation processes and inflammatory activation in case of diabetes are widely explored as potential mechanisms for influencing the cardiovascular risk.

Hyperglycemia, as a pathogenetic basis of diabetes, potentially contributes to tissue damage in several ways. When glycolysis is blocked, alternative pathways of glucose oxidation, in particular polyol and hexosamine, are activated. Activation of the polyol pathway leads to enhanced formation of reactive oxygen species, triggering oxidative stress (OS), which plays a key role in inducing smooth muscle cell apoptosis and cardiac remodeling [5-7]. Activation of the hexosamine glucose utilization way leads to increased transcription of inflammatory cytokine genes, which contributes to vascular inflammation and proatherogenic conditions. Hyperglycemia also activates the glycosylation process, which is accompanied by a cascade of complex biochemical reactions and leads to damage to macromolecules, alteration of their structural integrity, and impaired function. Advanced glycation end-products (AGEs), which accumulate in the tissues, lead to the formation of free oxygen radicals and enhance OS. When AGEs interact with their receptors, an entire cascade of signaling mechanisms is activated, which leads to increased expression and secretion of a number of proinflammatory cytokines, tumor necrosis factor-α (TNF-α), interleukin-1, and interleukin-6 (IL-6) [8].

Activation of the cytokine system plays a significant role in the pathogenesis of both metabolic disorders and coronary heart disease and is a marker of severity and a predictor of progression of these diseases [9, 10].

In light of the above, alpha-lipoic acid (ALA) is intriguing, considering the positive effects of the agent such as antioxidant properties, vasorelaxation, positive metabolic profile, as well as an anti-inflammatory potential [11, 12]. Moreover, the need of patients for ALA is determined by its deficiency in diabetes [13].

The purpose of this research is to study the dynamics of C-reactive protein (CRP), IL-6, TNF-α, and interleukin-10 (IL-10) in patients with type 2 diabetes who underwent non-Q-myocardial infarction (non-Q-IM), against the background of ALA.

## Material and Methods

### The criteria for involving patients into the research

One hundred twelve patients (67 men and 45 women, mean age – 61.79 ± 8.34 years with type 2 diabetes who underwent non-Q-MI) were examined. The control group (CG) was comprised of 40 almost healthy people matched by age and sex. The criteria of a healthy person were the absence of a history of chronic diseases, including diabetes and coronary heart disease, normal physical examination data, no abnormalities in the results of general clinical laboratory examinations, ECG and echocardiography, negative treadmill test.

Outpatients who were examined at the clinical base of the Fourth Department of Internal Medicine of the Bogomolets National Medical University (Kyiv, Ukraine) were involved in the research during 2015-2017.

The criteria for involving patients into the research were: 1) patients with type 2 diabetes with a history of non-Q-MI receiving oral antidiabetic therapy and basic therapy including an ACE inhibitor, a β-blocker, a statin, and an antiplatelet agent; 2) the patient's informed consent to participate in the research.

The criteria for not participating in the research were: 1) the presence of type 1 diabetes mellitus; 2) patients with type 2 diabetes receiving insulin therapy; 3) the presence of congenital or acquired heart disease, cardiomyopathy, persistent atrial fibrillation/atrial fluttering, symptomatic hypertension, heart failure III-IV functional class (FC), liver and kidney diseases, infectious and oncological pathology.

In the case of complete patient compliance fulfilling the criteria to be involved in the research and the absence of non-inclusion criteria, patients were divided into two groups: the main (n = 59) and the experimental group (n = 53). The distribution was carried out by random sampling. Patients in the main group were administered ALA 600 mg per os 30 minutes before breakfast in addition to the baseline therapy; instead, experimental patients received baseline therapy only. The duration of treatment and follow-up of the subjects was four months.

Before and after the treatment, the following were determined: the concentrations of CRP in the serum using the latex-turbidimetry method on a Cobas 6000 analyzer (Switzerland); the levels of proinflammatory cytokines IL-6, TNF-α and anti-inflammatory IL-10 in the serum by enzyme-linked immunosorbent assay using A-8768 IL-6-ELISA-BEST, A-8756 alpha-TNF-ELISA-BEST and A-8774 IL-10-ELISA-BEST produced by CJSC Vector-Best (Russia) on the analyzer HUMAREADER (Germany). Units for CRP are mg/L and for IL – pg/ml. Venous blood was collected from patients on an empty stomach (last meal >10 hours before blood collection).

### Statistical analysis

Descriptive statistics were used to describe data (means and standard deviations). After examining the normal distribution of data with the Kolmogorov-Smirnov test, a paired t-test was used (at a significant level of P <0.05) to examine the differences before and after the treatment with the assessment of the Standardized Mean Difference. Data were analyzed with STATA 12.

## Results

When comparing the baseline data for the investigated parameters of the patients with CG, an increase in the CRP concentration by 2.7 times (p <0.001), proinflammatory IL-6 (p <0.001) and TNF-α (p <0.001) by 4.4 and 3.1 times, respectively, as well as the anti-inflammatory IL-10 concentrations by 1.9 times (p <0.001) was revealed in patients of the main group. The patients of the experimental group also had significantly higher CRP concentrations (2.9-fold; p <0.001), IL-6 (4.2-fold; p <0.001), TNF-α (2.9-fold; p <0.001) and IL-10 (1.7-fold; p <0.001). The data obtained indicate the activity of systemic inflammation in patients with type 2 diabetes with non-Q-MI (Table 1).

**Table 1:** CRP and cytokine status in patients during the treatment (mean ± standard deviation).

Variables	Time	Main group (n=59)	Compare group (n=53)	Control group (n=40)
**CRP, mg/L**	Before	4.39±2.15#	4.68±2.36#	1.61±0.78
After	3.03±1.50	4.49±2.08
**IL-6, pg/ml**	Before	7.01±3.18#	6.58±3.47#	1.58±0.79
After	4.93±2.30	6.17±2.76
**TNF-α, pg/ml**	Before	6.47±2.39#	5.93±2.50#	2.06±0.99
After	5.00±2.08	5.85±2.11
**IL-10, pg/ml**	Before	6.03±2.03#	5.34±2.45#	3.20±1.20
After	5.52±1.86	5.26±2.18

Note: # – p <0.001 compared with CG individuals.

Analysis of the investigated parameters in the main group after receiving ALA for four months showed a significant decrease in the concentration of CRP by 30.9% (p <0.05), IL-6 by 29.7% (p <0.01) and TNF-α by 22.7% (p <0.01). There was also a tendency for IL-10 to decrease by 8.5% (p <0.2). There were no significant dynamics of the above parameters in the experimental group (Table 2).

**Table 2:** Assessment of the dynamics of Variables and Standardized Mean Difference (SMD).

Variables	Groups	Mean	95% Confidence Interval of the Difference	p	SMD (95%CI)	95% Confidence Interval of the SMD
			Lower	Upper			Lower	Upper
**CRP, mg/L**	Main group	-1.36	-2.04	-0.68	0.0001*	0.734	0.361	1.107
Compared group	-0.19	-1.04	0.67	0.661	0.085	-0.295	0.466
**IL-6, pg/ml**	Main group	-2.08	-3.09	-1.07	0.0001*	0.75	0.376	1.123
Compared group	-0.41	-1.62	0.80	0.502	0.131	-0.25	0.512
**TNF-α, pg/ml**	Main group	-1.47	-2.29	0.65	0.0005*	0.656	0.286	1.027
Compared group	-0.08	-0.97	0.81	0.859	0.035	-0.346	0.415
**IL-10, pg/ml**	Main group	-0.51	-1.22	0.20	0.157	0.262	-0.1	0.624
Compared group	-0.08	-0.97	0.81	0.859	0.034	-0.346	0.415

Note: P – Paired T-Test (before and after); * – Significant at P <0.05; SMD – Standardized Mean Difference (Cohen’s d) for repeated measures (<0.5 – ‘small’ effect size, 0.5-0.8 – ‘medium’ effect size, >0.8 – ‘large’ effect size).

**Figure 1:**
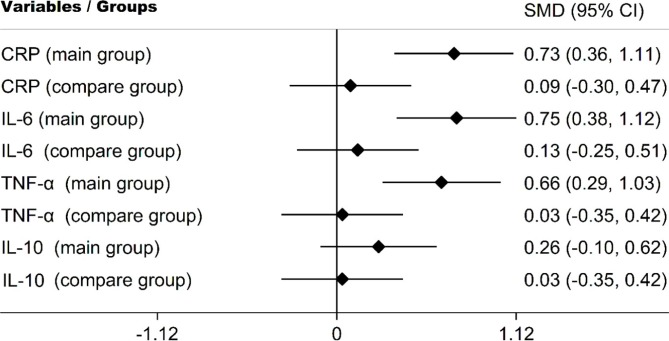
Standardized Mean Difference (effect size) in study groups.

Comparative analysis of treatment results confirmed significantly higher clinical effects in the main group compared to the experimental group. The standardized clinical effect size in the main CRP, IL-6, TNF-α group corresponds to the average clinical effect size and significantly exceeds the dynamics in the experimental group (Figure 1).

Therefore, as a result of the treatment using ALA, there was a decrease in the concentration of CRP and proinflammatory cytokines, which led to a decrease in the level of anti-inflammatory IL-10.

## Discussion

### Studying the anti-inflammatory activity of ALA

The clinical experience with the anti-inflammatory activity of ALA is limited to a few studies. Thus, in a study conducted by Zhang Y. et al., with a 2-week administration of ALA at a daily dose of 600 mg to 13 patients with obesity and impaired glucose tolerance, a significant decrease in IL-6 concentration of 16.7% and TNF-α of 24.6% was obtained [14]. In a multicenter, randomized, double-blind, placebo-controlled ISLAND investigation (Irbesartan and Lipoic Acid in Endothelial Dysfunction), it was also found that in the group of patients receiving ALA (n = 15) per os at a dose of 300 mg/day there was a significant 15% decrease in IL-6 levels within 4 weeks [15]. In the study of Hosseinpour-Arjmand S. et al., there was also a significant decrease in the serum concentration of IL-6 in obese patients with non-alcoholic fatty liver disease who received orally 1200 mg ALA per day for 12 weeks (n = 23) in comparison with the placebo group (n = 22) [16]. Other studies demonstrated that adding valsartan to an intravenous infusion of 600 mg of ALA once a day for 14 days in patients with early-stage diabetic kidney disease (n = 51) is beneficial [17–20].

Despite the somewhat contradictory results of the presented studies, most of these studies confirm the anti-inflammatory activity of ALA and coincide with the data obtained, although they were conducted on different, mostly small patient cohorts, with different doses, routes and terms of administration of the investigated drug.

### The possible ways of the anti-inflammatory effect of ALA

The analysis of the experimental base of in vitro [21-23] and in vivo [22, 24] studies revealed the possible ways of the anti-inflammatory effect of ALA. It's interesting that this effect is considered to be independent of the antioxidant activity of the drug [23]. In particular, the anti-inflammatory effect is explained by the ability of ALA to inhibit the proinflammatory transcription factor NF-kB by modulating the MAPK (mitogen-activated protein kinase) signaling pathway or restoring vitamin E with subsequent inhibition of protein kinase C, which is capable of phosphorylating IkF cytoplasmic 21B. Therefore, ALA is capable of inhibiting the NF-κB-induced transcription of a variety of molecules associated with inflammation, vascular adhesion, and monocyte migration, including proinflammatory cytokines. Experimental data from Ying Z. et al. confirm the activity of ALA in the atherosclerotic process [24]. According to the results of the research, ALA inhibited the vascular expression of NF-κB, translocated NF-κB from the cytosol to the nucleus and its activity, reduced T-cell (CD3+) infiltration in atherosclerotic plaques. Experimental work by Zhang W.J. et al. showed that ALA effectively suppresses the lipopolysaccharide-induced acute inflammatory response in vitro and in vivo by activating the phosphoinositol-3-kinase/protein kinase B (PI3K/Akt) signal path [22]. This path plays an important role as a negative feedback controller from an over-immune proinflammatory response. The results of another experimental work by Rousseau A.S. et al. were also interesting; it was found that ALA reduced the activation of another proinflammatory JNK signaling pathway that induces cell apoptosis [25].

Thus, the results of these researches provide an understanding of the possible mechanisms of the anti-inflammatory activity of ALA and are the basis for the substantiation of administering ALA to patients with type 2 diabetes mellitus and coronary heart disease.

Thus, according to the results of our research, taking ALA for 4 months in patients with type 2 diabetes who underwent non-Q-MI reduced the activity of systemic inflammation and did not significantly affect the content of anti-inflammatory IL-10 in these patients. However, further randomized, placebo-controlled studies are required to confirm these results.

## Conclusion

IM patients with type 2 diabetes (main group) have a 2.7 times increase in CRP content, 4.4 and 3.1 times in proinflammatory IL-6 and TNF-α, respectively, as well as anti-inflammatory IL-10 concentration of 1.9 times higher relative to the indicators in the control group.

Administering ALA for 4 months to patients with type 2 diabetes mellitus on the background of basic therapy contributes to a significant reduction in CRP by 30.9%, IL-6 by 29.7%, and TNF-α by 22.7%.

Hence, ALA, in addition to the basic therapy in those patients, can be used to reduce the activity of systemic inflammation as a predictor of DM and CHD progression.

## Conflict of Interest

The authors confirm that there are no conflicts of interest.
